# Supplementary material for: Circulating Tumor DNA for Prediction of Complete Pathological Response to Neoadjuvant Radiochemotherapy in Locally Advanced Rectal Cancer (NEORECT Trial)
Source: Cancers (Basel). 2024 Dec 14;16(24):4173. doi: 10.3390/cancers16244173 (PMC11674684; doi:10.3390/cancers16244173)
Supplement: Supplementary file 1 [file cancers-16-04173-s001.zip › cancers-3356568-supplementary.pdf]

Supplementary Materials:

Supplemental table S1: Basic patient and tumor characteristics of the intent-to-treat population

Intent to treat (n=40)		
**Age**		(%)
Median (Range)	68 (37-87)	
**Sex**		
Male	29	73
Female	11	28
**cm a.a. (min)**		
Median (Range)	7 (0,1-11)	
**cT**		
2	2	5
3	29	73
4	9	23
**cN**		
0	6	15
1	11	28
2	17	43
+	5	13
x	1	3
**G**		
2	30	75
3	2	5
x	8	20
**cM**		
0	34	85
1	6	15

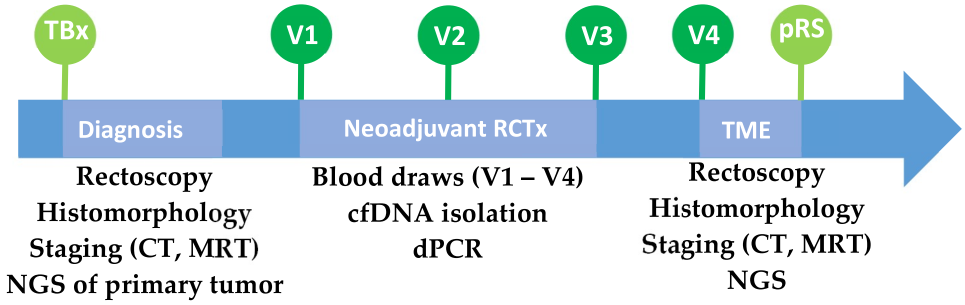

Supplemental Figure S1: Overview of the NEORECT trial. At the time of diagnosis, the tumor is assessed in the context of routine diagnostics by rectoscopy and MRT. A tissue sample is also obtained for histomorphological classification and molecular analysis. During neoadjuvant treatment (nCRT) until surgery (TME) four blood draws (V1 – V4) will be conducted for cfDNA isolation and ctDNA analysis by dPCR. A second rectoscopy and staging will be conducted in the context of surgery and histopathological remission status will be defined by the pathologists.
cfDNA: cell-free DNA; dPCR: digital PCR; RCTx: Radiochemotherapy; pRS: pathological remission status; TBx: Tissue biopsy

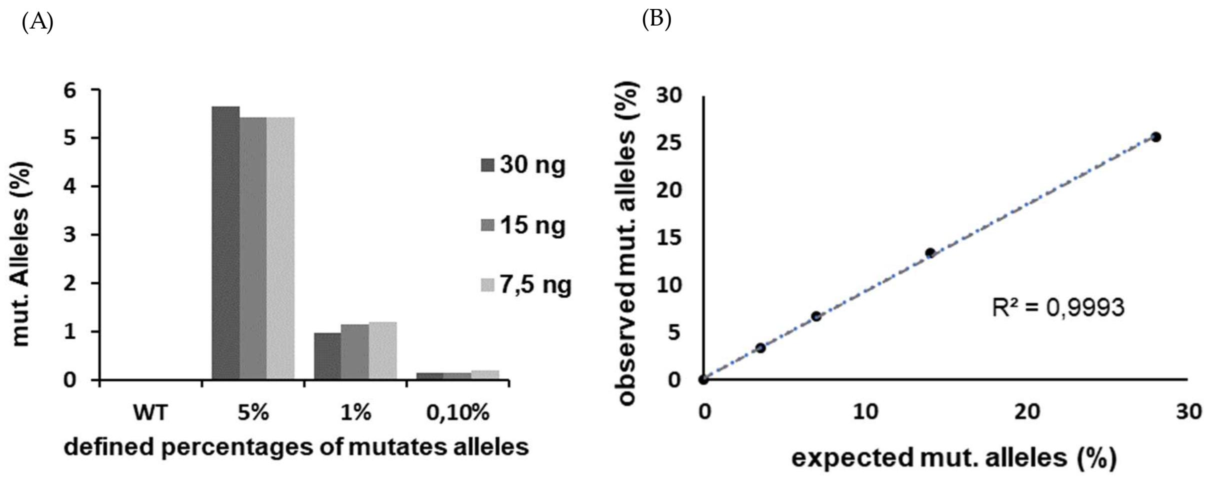

Supplemental Figure S2: Testing the limit of detection (LoD) and reproducibility of dPCR

A- Defined percentages of the *EGFR* mutation T790M were analyzed by dPCR with different DNA inputs. The results are shown as detected percentages of mutated alleles (y-axis).

B- Correlation of the observed and expected results of a dilution series of *KRAS* positive cfDNA with wildtype cfDNA as measured by dPCR (Input: 1200 GE/ ml plasma).

Mut.: mutated; WT: wild-type (DNA)

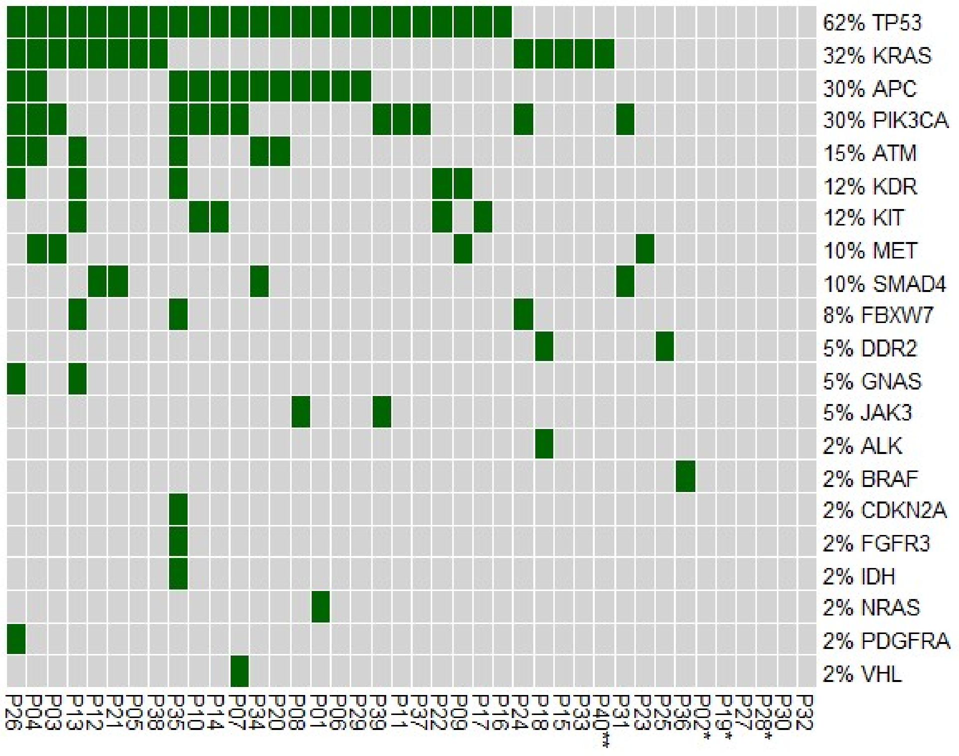

Supplemental Figure S3: Detected mutations by next generation sequencing of all patients included into the trial (n=40). Twenty-one mutations were detected in total by NGS. Between 2% and 62% of the samples were positive for each mutation within the NeoRect cohort of 40 patients.

* no NGS data available
